# Rail Transport Delay and Its Effects on the Perceived Importance of a Real-Time Information

**DOI:** 10.3389/fpsyg.2021.619308

**Published:** 2021-06-16

**Authors:** Mahdi Rezapour, F. Richard Ferraro

**Affiliations:** ^1^WYT2, Department 3295, Laramie, WY, United States; ^2^Department of Psychology, University of North Dakota, Grand Forks, ND, United States

**Keywords:** structural equation modeling, stress, delay, physical effects, real-time information, passenger information system, public transport

## Abstract

Various psychological feelings that commuters might experience due to the shortcomings of a public transport are a major concern for transport policy makers. Those shortcomings would be translated into various negative psychological feelings, which would consequently tarnish the perceived quality of the public transport system in terms of its characteristics, e.g., the perceived quality of the passengers' information system (PIS). A delay has often been defined as the difference between the real arrival of a transport and the scheduled arrival of based on the PIS. The main question this study seeks to answer is how passengers view the PIS while undergoing various psychological negative impacts due to delay? This is especially important when the PIS is not precise. Previous studies on the importance of real-time information mainly focus on the impact of PIS on the satisfaction of commuters, or the reliability of the public transport. However, they rarely consider the negative psychological impacts that delays might have on commuters, and how those negative feelings might be aggravated by providing inaccurate information for the commuters. The proposed study is based on completed questionnaires by 396 passengers waiting for a rail transport in Malaysia; the rail transport was experiencing frequent long delays due to various mechanical malfunctions. In addition, the PIS provided for the passengers were mainly imprecise, and was updated regularly. The relationship between various considered variables, and a related latent factor, were formed by means of factor analysis. The results of internal consistency and validity highlight acceptable factors to be considered for a structural equation modeling (SEM) model. Three latent factors were found to impact the latent factor of PIS. For instance, it was found that the relationship between motion sickness factor and the response of PIS is not by a direct relationship between those two factors, but through the mediation of a latent physiological factor. On the other hand, the impact of the psychological feelings of the commuter by PIS is higher than its physiological effects. The results of this study have an important managerial implication for policy makers that even if the delay is inevitable, an accurate PIS could be provided to reduce the associated negative feelings of delay. Extensive discussion has been had about identification of a best fit model and process of model's parameters' estimation.

## Introduction

Public transportation systems are a crucial staple in society. Their benefits include a reduction in congestion, gasoline consumption, and carbon emissions, consequently creating safe and clean air. As a result, extensive efforts have been made by public transport planners to enhance the satisfaction of public transport users. That has been achieved, for instance, by an improvement in the quality of a certain service, such as reliability of the public transport arrival, which could lead to a greater use of public transport.

However, despite the efforts made toward improving public transport, the service is not without shortcomings in many parts of the world. One of the aspects of public transport which has received much attention its delay. Delay can be defined as a part of waiting time, being the difference between the actual arrival of a public transportation and the expectation of a commuter on the other hand. A commuter's expectation may be formed by time schedules presented by policymakers or real–time information display. However, if none of these exist, or no precise information is provided, it could be gained from the past experience of a commuter about average arrival or departure time of a public transportation system.

Delays are a major reason why commuters would be dissuaded from using a public transportation system. Thus, transport companies exert efforts to avoid those delays and improve punctuality. The fewer delays commuters face, the more likely commuters are to choose that service instead of other modes of transport (Jansson, 1993). However, sometimes delay is inevitable, so it is important to know how the delay is transferred to the commuters in terms of various behaviors, and how those feelings impact the perceived quality of the transport system. This is especially important as it is expected that the impact of delay on the commuters' perceived quality of the service will result in emotional or physical behaviors that the commuter might experience. Understanding those feelings not only inspire the companies to prevent them but could also help them to find a solution to improve their services, even if they cannot reduce the amount of delay.

As delays on public transport systems are often inevitable, extensive efforts have been made by transit planners and researchers to enhance the performance of public transportation systems. One of the main approaches is to provide passengers with a passenger information system (PIS) to increase the reliability of the commuter, and also reduce the commuters' uncertainty. It is believed that the movement of vehicles and passengers are inherently time-dependent (Hickman and Wilson, 1995). However, as the vehicle moves in a stochastic environment due to various factors, such as malfunction of the vehicle, the vehicle might not perform on schedule. Thus, real-time information could be offered to relieve commuter uncertainty. The PIS provides the passengers with information in real-time by displaying announcements about the arrivals of public transport.

Extensive research has been conducted on the importance of real-time information in the reduction of uncertainty about the public transport's arrival times (Balogh and Smith, 1992). Studies have also been carried out on a real-time bus arrival information system (Wepulanon et al., 2018), and real-time information systems' effect on the waiting experience of public transport users (Kellermann, 2017). The impact of public transport real-time information on customers was evaluated in the previous study (Dziekan and Kottenhoff, 2007). The positive psychological factors of real-time information were highlighted in that study. Those include factors such as reduced uncertainty, greater feelings of security, and higher customer satisfaction due to real-time information.

However, sometimes despite having an unreliable public transportation, the PIS is also unreliable, showing imprecise arrival times of public transport. For those scenarios, it is worth investigating the impact of inaccurate information of PIS on the psychological feelings of commuters, and how it impacts the quality of that public transportation system.

It is expected that PIS has an effect on the perceived quality of a public transportation by means of relieving various negative psychophysical feelings of the commuters due to the uncertainty associated with delay. Although the importance of real-time passenger information systems has been highlighted in the literature review, it is still unclear how the positive impacts of the real-time, or even the negative impacts of inaccurate real-time information, would be translated to the passengers. For the case study used in this study, there is uncertainty about the arrival of rail transport.

In this study, the PIS was provided for the commuters by means of an LCD display at the terminal of the station. However, as discussed, a reliable measure of the rail transport arrival was not provided for commuters by means of the PIS so there was uncertainty for both the arrival of the train and also PIS accuracy.

### Problem Statement

It is hypothesized that delay is not only a matter of slowing down the time it takes for a commuter to reach a destination, or the monetary value of time lost, but an issue of well-being for commuters. Even a monetary valuation of time itself could be translated into behaviors of the commuters. Thus, the impact of delay on the commuters could be looked at from a psychological angle, such as stress or anxiety experienced, as well as the cost of the psychological behaviors of people.

For instance, stress could lead to serious illnesses such as cardiovascular illness and suppressed immune functioning (Wener et al., 2005). Various feelings could be translated from stress such as feelings of anxiety, fear, or even anger. For instance, delay was highlighted as a main source of stressfulness when traveling by car, especially through traffic (Wener et al., 2003). Crowding, delay, and accessibility to a railway station were some of the sources of commuters' anxiety (Cheng, 2010).

Despite the fact that the presence of delay is often inevitable, public transport experts have done their best to reduce the uncertainty felt by commuters by providing measures such as real-time information, so the commuters would be informed about a possible delay, and expected time of the arrival of the train. That would mitigate the translated negative effects of delay on commuters.

However, despite the importance of real-time information, often the information is inaccurate and imprecise. It is hypothesized that inaccuracy of the real-time information would impact not only the perceived level of various psychophysical factors that the commuters would experience, but also as a result of those factors, it would tarnish the perceived quality of the rail transport system. Thus, this study is conducted to unlock the relation of various translated feelings that the commuters might experience, and to investigate the effects of those feelings on the perceived quality of the transport through real-time information.

## Data

This section will be presented in two subsections. The first subsection will outline the design of the questionnaire, while the second subsection will give an overview of various instrument's explanatory variables.

### The Instrument

**Figure 1:**
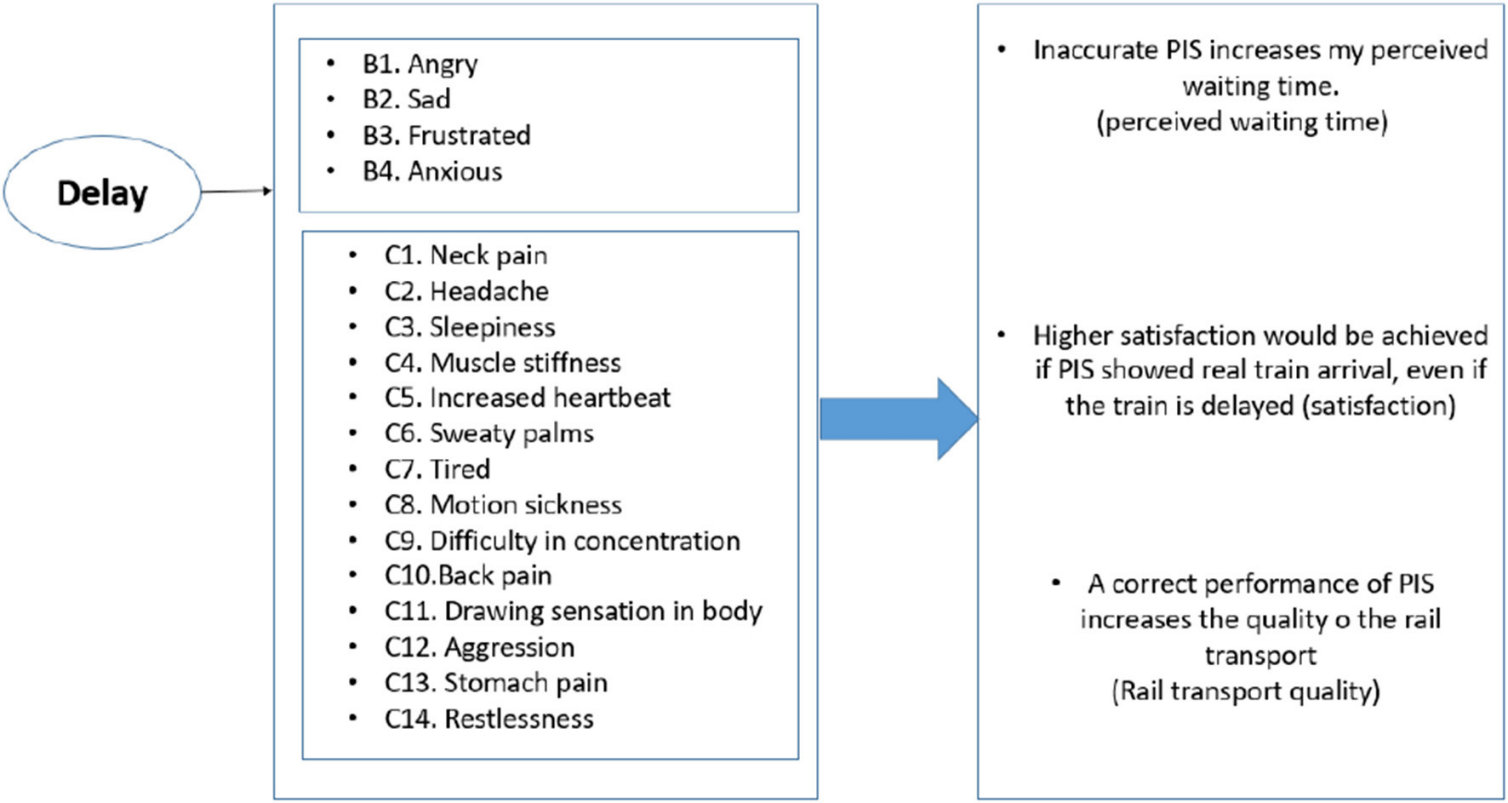
List of questions included in part B and C of the instrument, and considered questions in part D.

In the questionnaire, the commuters were asked to indicate to what degree they agree they experience various emotional or physical feelings when facing rail transport delay. On the same scale, in the last part of the questionnaire, they were asked over five questions, on the same scale, about the impacts of delays on various aspects of the transport system, e.g., satisfaction or quality. The first three questions were about PIS and its impact on the perceived waiting time, and the impacts of PIS on their satisfaction and perceived quality of the rail transport system (see Figure 1). Various feelings were used as a proxy for the delay, and then those feelings were used as factors to structure the degrees of various beliefs about the public transport system.

The questionnaires were distributed to 419 commuters at the station of Serdandg, which is one of the main stations of Keretapi Tanah Melayu (KTM) in Malaysia. The surveys were distributed during off-peak hours from 4 to 7 pm to be consistent in our evaluation. Questionnaires were translated into the local language, Malay, by a Malaysian PhD student in the field of education. The questionnaire had an introduction explaining the objective of the study, and various sections of the questionnaires that the commuters were expected to answer. The respondents were requested to leave the questionnaire blank if they were not interested.

The instrument had four parts: background (nine questions), psychological effects (four questions), physical effects (14 questions), and general questions (five questions). All questions except for the first part were based on a 5–scale question type. Due to the design nature of the study, the first part of the questionnaire was excluded from the analysis. Among 419 distributed questionnaires, 396 of them were completed and used for the analysis (a response rate of 94%).

It was noted that due to the satisfying behavior of respondents, some of the responses would result in incomplete or biased information retrieval (e.g., choosing the first response alternative), or no information retrieval (Krosnick, 1991). A solution has been proposed by giving an alternative of “I do not know” or “undecided” instead of reporting an opinion. As a result, we incorporated in all instruments questions, except for the first part, an alternative of “undecided.” An undecided answer could be considered as similar to a middle response (Groothuis and Whitehead, 2002). To evaluate the feelings that the commuters might feel due to delay, the respondents were asked in the questionnaire statements such as “I feel angry when I face delay in KTM” and the respondent would answer on a scale of 1–5, from “1-strongly agree” to 5-“strongly disagree,” the degree of their agreement.

The physical section of the survey was based on the Cohen-Hoberman inventory of physical symptoms (CHIPS) (Cohen and Hoberman, 1983). That is a list of 39 common physical symptoms highlighting a relationship between negative life stress and various physical symptomatology. Those includes factors such as back pain, diarrhea, and headache. The factors were filtered to include only 14 variables based on our case study. Again, all the effects are based on the reviews of the literature.

Various sources were used for the design of psychological aspects of the questionnaire. A self-report measure of stress was developed and tested (Greller and Parsons, 1988). The scale included various physiological and psychological descriptors. Psychological factors include factors such as being angry, nervous, or stressed. Some of the physical factors included neck pain and feeling tired. The design of this part of the survey was also similar to the previous study, which was conducted to illustrate the capability of a cognitive-motivational-relational theory for predicting emotions (Lazarus, 1991). For that study, 15 different emotions were identified including negative emotions such as anger, anxiety, sadness, and disgust.

### Data Descriptions

A total of 396 fully completed responses were collected and considered for the analysis. In section B and C, the respondents were asked on a 5-scale Likert questions about the feelings that they might experience while facing delay. The scale had the following alternatives: Strongly agree (1), agree (2), undecided (3), not agree (4), and strongly disagree (5). For instance, based on Table 1, feeling frustrated and angry were some of the feelings that respondent reported experiencing the most while facing delay when waiting for the rail transport. The initial examination of the data reveals that, as expected, an overwhelming majority of the respondents rated the impact of delay very negatively, and in favor of various emotional or physical feelings. More descriptions about the predictors is presented in Figure 1.

**Table 1:** Descriptive summary of important factors and response.

**Variables**	**Mean**	**Variance**	**Min**	**Max**
**Response**				
D1, I experience increased perceived waiting time due to imprecise PIS	1.91	0.701	1	5
D2, I would experience increased satisfaction in the case of accurate PIS	1.843	0.99	1	5
D3, I would experience an increase in perceived quality of the rail transport service due to accurate PIS	1.649	0.552	1	5
**Predictors**				
**Psychological feelings**				
B1, being angry	1.840	0.868	1	5
B2, being sad	2.50	1.410	1	5
B3, being frustrated	1.874	0.971	1	5
**Physical feelings**				
C8, feeling motion sickness	3.212	1.348	1	5
C13, feeling stomach pain	3.306	1.236	1	5
**Physical feelings**				
C1, Neck pain	2.306	1.292	1	5
C2, headache	2.669	1.307	1	5
C4, muscle stiffness	2.230	1.220	1	5
C10, back pain	2.248	1.280	1	5
C11, sensation in body	1.977	1.060	1	5

Regarding the sample size, a few points need to be highlighted. Various values as the minimum sample size have been highlighted in the literature review. For instance, a value of 500 was considered for the sample size for providing accurate parameter estimates (Jiang et al., 2016). However, it has also been discussed that there is no golden rule for the number of required samples (Morizot et al., 2009). In addition, while 500 was recommended for an ideal condition for accurate parameter estimate, the model could still be estimated successfully with 250 respondents (Reeve and Fayers, 2005).

When conducting any survey, there might be some associated bias that should be taken into consideration. That could include sampling bias or bias in how the respondents were selected. Regarding this concern, we asked any passenger waiting for the rail transport whether they were interested in participation in our survey. No inclination was given to any age/sex groups while collecting the dataset.

Regarding the nonresponse bias, we excluded questionnaires that were left unfilled. These remained even though we included the middle option for uninterested commuters. In summary, we considered the possible biases and tried to minimize their impact (for instance, by inclusion of “I do not know” or “undecided” as one of the answers to our questionnaire).

Finally, it should be noted that another study in the literature review used a similar case study used in the current manuscript. Rail passenger crowding was evaluated with the help of factor analysis and structural equation modeling (SEM) in the literature review (Mohd Mahudin et al., 2012). The study came up with an instrument to capture crowding and links it with an experience of feeling stress and exhaustion. Feeling irritable, stressful, frustrated, tense, and unpleasant were some of the factors that were considered in that study.

## Method

As the factor analysis (FA) is an initial step in conducting the SEM, first this section will discuss the FA and its implementation, and then it will go over the process of the SEM.

### Estimation of Factor Analysis

The objective of the FA is to provide insight about latent variables behind the observations' behavior. In other words, factor analysis would be used to reduce the data complexity by identifying fewer factors explaining the data. Here the data complexity reduction could be achieved by the process of singular value decomposition (SVD). The objective is to approximate the R matrix of rank n with a matrix of lower rank, R, which here could be written as: (Revelle, 2014).

(1) $$\begin{array}{l} R=I_{sd}CovI_{sd} \end{array}$$

where *I*_*sd*_ is a diagonal matrix with the element of 1/sd*i*, $sd=\sqrt{diag\left(Cov\right)}$, and $Cov=\frac{XY}{N}$, where X and Y are two matrices that are used to calculate the Cov.

One of the main aspects of factor analysis is its loadings, which explain the correlation across the main variables and unseen components extracted by the analysis. The objective of conducting factor analysis is also viewed as reducing matrix dimensionality by looking at variables that correlate highly with the latent group, and correlate badly outside of that group (Field, 2009).

A factor would be called as those variables with high inter-correlation that could well measure the original variables. On the other hand, the approximated correlation matrix (R) could be seen by the product of the two factors to be summed by a diagonal matrix of uniqueness (*U*^2^), as follows:

(2) $$\begin{array}{l} R\sim FF^{T}+U^{2} \end{array}$$

where F and *F*^*T*^ are factor and its transpose, respectively, and U is a diagonal matrix of uniqueness. R is similar to the matrix of correlation and would be used to approximate the correlation matrix. Here, the objective is to approximate the correlation matrix based on some factors and Us.

The equation above would be solved by considering simultaneous equations by unweighted least square (ULS), which minimizes the sum of the squared residuals when considering the correlation matrix from above and the covariance matrix of S (Revelle, 2004):

(3) $$\begin{array}{l} E=0.5trace{\left(S-R\right)}^{2} \end{array}$$

where trace is the sum of the diagonal elements of the matrix, S is a sample covariance matrix, and R was defined before. The above would be solved by giving starting values to matrices and using an appropriate optimization function for the above equation to be minimized. The solution would be achieved by iterative methods, finding a best fitting solution.

After the optimization process is employed from the above, rotation would be conducted. As factor extraction is difficult to interpret, rotation would be used to provide slightly different axes. Oblique rotation assumes that the factors are correlated, while orthogonal rotation assumes that the factors are uncorrelated. The objective of rotation is to achieve a simple structure (Jennrich and Sampson, 1966), and a matrix is said to be rotated if a multiplication by orthogonal vector preserves the communalities of variables (Revelle, 2014). For orthogonal, although the factors would be uncorrelated, the correlation across factors exists for oblique transformation. Variables *x*_*i*_ could be written as a set of weighted linear sum as follows:

(4) $$\begin{array}{l} x_{i}\sim \overset{n}{\underset{j=1}{\sum }}w_{ij}F_{j} \end{array}$$

The above is a set of regression weights or loadings. In this study, as we had a small sample size and there is a great chance of correlation, oblique rotation was used for identification of the factors. In obliques rotation, new axes are not required to be orthogonal.

The squared multiple correlation (SMC) is an important aspect of the FA, which is the initial estimate of a variable communality, highlighting the proportion of the total variation explained by the model, and factors holding the highest variability would be highlighted. The SMC would be estimated by 1 – 1/diag(*R*^+^) where *R*^+^is the inverse of R.

To have an inverse of R, the Moore-Penrose Pseudo Inverse would be used as sometimes the matrix is less than full rank. For that, first the singular value decomposition (SVD) of the matrix would be employed (udv matrix). The SVD is a factorization of a real or complex matrix that generalizes the Eigen decomposition of a square normal matrix to make certain subsequent matrix calculations simpler. That is a product of three matrices, where d is Eigen values vector and u and v are Eigen values matrices. Considering *A* = *U*Σ*V*, the inverse would be estimated by *A*^+^ = *V*Σ^+^U^*^, where + is an inverse of a matrix, and ^*^ is its transpose.

In summary, the factor, latent variable, and model could be written in a form of (Browne and Arminger, 1995):

(5) $$\begin{array}{l} x=\mu +\Lambda z+u \end{array}$$

where x is p×1 of manifest (observed) variables, z represent m×1 common factors, u represent p×1 unique variables, Λ, p×m is the factor matrix of partial regression weights of the manifest variables on the factors or factor loadings, and elements of μ are corresponding intercepts.

### SEM

Now the factor analysis could be extended by incorporating linear structural relations across factors. Consider two factors of *z*_*x*_, *z*_*y*_ are identified through factors analysis, the relations between these two factors should satisfy the linear structure of

(6) $$\begin{array}{l} z_{y}=B_{y}z_{y}+\Gamma z_{x}+e \end{array}$$

where *B*_*y*_ and Γ are regression weights or path coefficients, and elements of *z*_*x*_ that do not depend on other variables (exogenous), while elements of *z*_*y*_ depends on *z*_*x*_ through elements such as Γ, being endogenous, and “e” represents error terms.

Path analysis is considered as a special form of the SEM. While path analysis assumes that variables are measured with no error, the SEM uses latent variables to account for the measurement error. The latent variable alone could be seen as causes of a few observed behaviors. In other words, it assumes that the correlation between variables, including errors, would be 1. However, while connecting the path between factor and explanatory variables, the weights of paths would vary. The multiplication of the two paths would be a correlation across various variables, and the differences would be set as error.

Manifest (observed) variables, which measure the endogenous factors, would be represented by y, while those factors measuring exogenous factors would be represented by x, and those factors could be written based on their relevant parameters based on the above factor analysis equation.

Now the covariance structure of $\Sigma =\left(\begin{array}{ll} \Sigma_{yy} & \Sigma_{yx}\\ \Sigma_{xy} & \Sigma_{xx} \end{array}\right)$ would be written as (Browne and Arminger, 1995):

(7) $$\{\begin{array}{c} \Sigma_{yy}=\Lambda_{y}{\left(I-B_{y}\right)}^{-1}\left(\Gamma \Phi_{x}\Gamma^{\prime }+\Psi \right)\;{\left(I-\;B_{\;y}^{\prime }\right)}^{-1}\;\Lambda_{\;y}^{\prime }+\Theta_{u_{y}}\\ \Sigma_{yx}=\Lambda_{y}{\left(1-B_{y}\right)}^{-1}\Gamma \Phi_{x}\Lambda_{\;x}^{\prime }=\;\Sigma_{\;xy}^{\prime }\\ \Sigma_{xx}=\Lambda_{x}\Phi_{x}\Lambda_{\;x}^{\prime }+\Theta_{u_{x}} \end{array}$$

where Θ_*u*_*y*__ *and* Θ_*u*_*x*__ are covariance matrices where $\Theta_{u_{y}}=cov\left(u_{y},{u^{{\;}^{\prime }}}_{y}\right),\;and\;\Theta_{u_{x}}=cov\left(u_{x},{u^{{\;}^{\prime }}}_{x}\right)$, Λ_*x*_and Λ_*y*_ contain regression weights for manifest variables on exogenous and endogenous variables, respectively. *B*_*y*_ on the other hand, includes weights on other endogenous factors. Γ Includes regression weights of endogenous factors on exogenous factors. Φ_*x*_, ψ, Θ_*y*_, Θ_*x*_ are covariance matrix for exogenous, covariance matrix for regression errors, unique variance corresponding to endogenous factors, and unique variance corresponding to exogenous factors, respectively. The above formulation is based on LISREL (Diamantopoulos and Siguaw, 2013).

However, the LISREL has been expanded to the Reticular Action Model (RAM) (McArdle and McDonald, 1984), being the same model as LISREL with an emphasis on Σ^−1^. Now the RAM model, which this study is based on, could be written as (Brown and Mels, 1990).

(8) $$\begin{array}{l} \nu =\;B_{V}\nu +\nu_{x} \end{array}$$

Here ν includes all variables including manifest, factors, and errors variables. *B*_ν_ matrix is related to weights. While some rows of *B*_ν_ would be null, diagonal elements of *B*_ν_ would be zero. ν_*x*_includes exogenous variables along with their error terms in ν, while other endogenous were filled with zeroes. Now the covariance matrix of all variables of RAM data model could be written as (Browne and Arminger, 1995):

(9) $$\begin{array}{l} \gamma =cov\left(\upsilon ,\upsilon^{\prime }\right)={\left(1-B_{\upsilon }\right)}^{-1}\Phi_{\upsilon }{\left(1-B_{\upsilon }^{{\;}^{\prime }}\right)}^{-1} \end{array}$$

where $\phi_{\upsilon }=cov\left(\upsilon_{x},\upsilon_{x}^{\prime }\right)$, and all other parameters were defined before. Also, from the above equation, the covariance structure of the manifest variables could be written. In summary, an optimization program would be conducted on RAM's two matrices, and based on some discrepancy function the model would be optimized.

The discrepancy function is another aspect of SEM which needs some elaboration. The discrepancy function of SEM is a mathematical function highlighting how close a structural model conforms to observed data. Various discrepancy functions could be used as an objective function. An iterative process would be used, for instance, for minimizing the discrepancy function of generalized least squares as:

(10) $$\begin{array}{l} F_{GLS}\left(u,\;\eta \right)={\left(u-\;\eta \right)}^{\prime }V^{-1}\left(u-\;\eta \right) \end{array}$$

where V is positive definite weight matrix, *u* = $\left[\begin{array}{c} ȳ\\ \omega \end{array}\right]$, ω = *vecs*(*W*), and η = $\left[\begin{array}{c} \mu \\ \sigma \end{array}\right]$

where *y* is N×p represents a sample of N dependent observation; for the above the discrepancy function is zero if *y* = μ *and* ω = σ. The model would be employed by creating random starting values and updating them. Diagonally weighted least squares (DWLS) would be used for more accurate parameter estimates of the parameters where multivariate normality is severely violated, and as the data are ordinal, not following the assumption of multivariate normality.

### Model Goodness of Fit

Various goodness of fit could be used to evaluate the goodness of fit of the SEM. It should be noted that it is not just about what measure to use but also what cut-off to use for evaluation. Root mean square of approximation gives information about how well the factors fit the population covariance matrix (Byrne, 2013). A cut-off limit of 0.07 is recommended in the literature review for this measure (Steiger, 1990). Goodness-of-fit (GFI) estimates the proportion of variance being accounted by the estimated population covariance (Tabachnick and Fidell, 2001).

While this value ranges from 0 to 1, a cut-off of 0.9 is recommended for this fit (Shevlin and Miles, 1998). On the other hand, the root mean square error of approximation (RMSEA) was used by measuring the discrepancy between the hypothesized model, with optimally chosen parameter estimates, and the population covariance matrix. The standardized root mean square residual (RMSEA) is the most informative and popular method for comparison of various SEM models. RMSEA in the range of 0.05 and 0.10 was considered as fair fit values, while values above 0.10 indicates poor fit (MacCallum et al., 1996).

## Results

This section will follow the methodology structures: first the results of the factor and SEM models will be discussed, and then a few considered models will be compared. Recall, the questionnaire was distributed across 419 commuters waiting for rail transport, and 396 filled questionnaires were used for the statistical analysis.

### Factor Analysis Results

**Figure 2:**
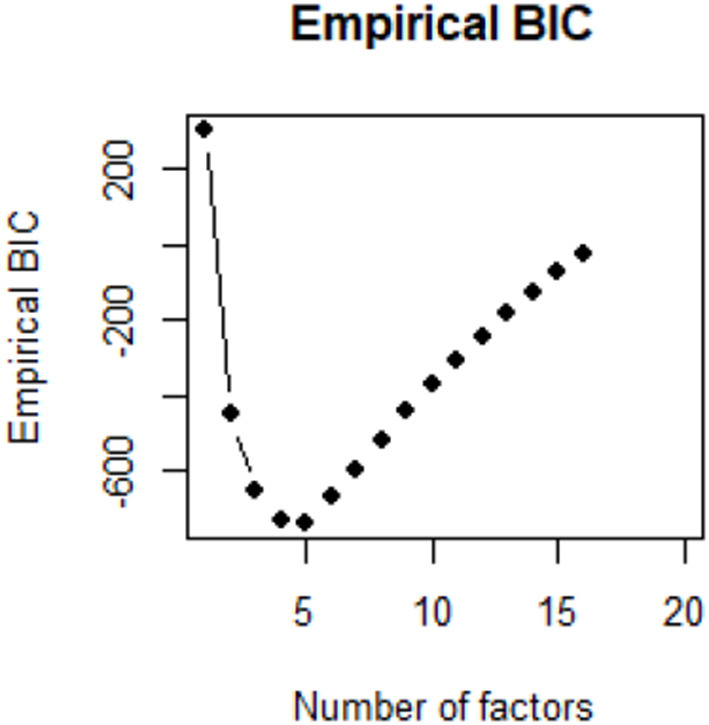
Measure for the number of factors.

While running factor analysis, it is important to consider the number of factors that will be used. For the factor analysis, we chose four factors due to a better fit, based on Bayesian information criterion (BIC), and ease of interpretability (see Figure 2). The factors were organized and named to be used for the SEM model. The measure was chosen based on BIC for each number of factors. That is BIC based on empirical chi square, eChiSq. eChiSq is based on observed residual correlation matrix and the observed sample size for each correlation.

The variables were considered as a factor if they had a loading >0.6, which the following paragraphs would outline. Motion sickness and upset stomach were highlighted as one factor. Association between motion sickness and upset stomach was confirmed by the past study by the use of the 5-point scale (Reason and Brand, 1975). In that study, the symptoms were measured immediately after rotation and once again 2 h later.

Motion sickness was also evaluated as a stress response, and was correlated with stomach issues (Money, 1996). The two variables were used to call the latent factor of “motion_sickness.” The impact in the literature was defined as they might impact the relative balance of endolymphatic and perilymphatic pressures in the labyrinth (Parker et al., 1983). That could be considered as the sympathetic nervous system which increases blood flow in skeletal muscles accompanying motions sickness (Johnson et al., 1993).

The other factor includes neck pain, headache, muscle stiffness, back pain, and drawing sensation in body. These factors were called “physioloocal_effects.” On the other hand, three of the psychological symptoms were found to be linked together, namely being angry, sad, and frustrated. Those are three out of four included psychological effects, except for being anxious, that we considered for our survey. Those would be referred to as psychological_impacts of delay.

For the response, five questions for the last part of the questionnaire were considered in the initial FA analysis. Three questions related to the PIS were found to be categorized under one factor. The questions categorized under the “PIS” all related to that item. The parameters were discussed in a previous paragraph (see Figure 1). Cronbach's alpha was used to check for consistency reliability. All the factors were checked to see if removing any predictors would result in an improvement in the value of Cronbach's alpha. The standardized Cronbach's alpha is computed as

(11) $$\begin{array}{l} \alpha_{s}=\frac{p.\bar{r}}{1+\left(p-1\right).\bar{r}} \end{array}$$

where p is the number of items in a latent and *r* is the average of all Pearson correlation coefficients between the items. As removing any of the considered variables in any factor did not result in an improvement in Cronbach's alpha, all the variables were kept in the factors. Also, Cronbach's alpha for all factors was at least 0.8.

### Model-Fit Comparisons

Before moving forward with the model results' interpretation, it is worth discussing how we came up with the final model. In this study, various scenarios were considered and checked to come up with a better model that could be comparable with the original data structure. For instance, we considered the residual variances of the two observed variables of feeling angry and feeling stressed to be correlated. We did that as it was hypothesized that those two predictors have something in common that could not be captured by a latent variable. For finding the solution, the factor's loading for the first indicator would be fixed to 1. However, other loadings are free, and their values would be estimated by the model.

For all the considered models, the response or dependent factor was set as PIS. The objective was to try various possibilities of connecting various feelings of the commuter to that dependent factor. The base model was considered as model number 1 in Table 2, where all the psychophysical latent factors would be connected to the latent factor of PIS directly.

**Table 2:** Comparison across some of the considered models.

**#**		**RMSEA < 0.08**	**GFI > 0.9**	**SRMR < 0.08**
1	Direct relation from physical and psychological symptoms to the PIS	0.086	0.987	0.065
2	Psychological effects through the mediating impact of physical effects	0.104	0.983	0.074
3	Physiological through the mediating impact of motion sickness	0.094	0.986	0.071
4	Physical factors are all as one factor	0.118	0.979	0.080
5	Motion sickness through the mediating impact of physiological impacts	0.117	0.978	0.082
6	Impact of motion sickness through the mediating impact of physiological impacts	0.080	0.924	0.055

The other models were considered as an extension of the first model. Various mediating effects were considered to see if the impacts of various latent factors on the PIS could be through some mediating path. For instance, it was checked to see if the impacts of psychological feelings on PIS could be through the mediating effect of physical aspects of the delay on the commuters. In summary, the two models 1 and 6 were found to perform the best. Although it might be clear from the results in Table 2 that the sixth model might perform better than the first model, the comparison was confirmed by analysis of variance (ANOVA) test.

The ANOVA was conducted to test if the difference is significant across the two models. The results highlighted that the chi-square of the sixth model is substantially smaller than the first model (better fitting) confirming that the sixth model is better in representing the sample data (DF diff = 4, *p*-value < 0.05, chi-square diff = 65).

It should be noted that the weighted least squares means and variance adjusted (WLSMV) were used for parameters' estimations. It has been discussed that the WLSMV does not assume normally distributed variables, and it would provide a best option for modeling ordered data (Brown, 2015). It should also be noted that all the values are standardized parameters (completely standardized solution) so comparison could be made across parameters' estimated values.

### Structure of the Finalist Model

The question is what factors impact the feelings of passengers, and consequently their perception about the PIS. What are the passengers' feelings that impact the importance of PIS, assigning more importance on the quality and satisfaction of the rail transport based on PIS? The following section will detail the underlying relationship between the identified structures.

**Figure 3:**
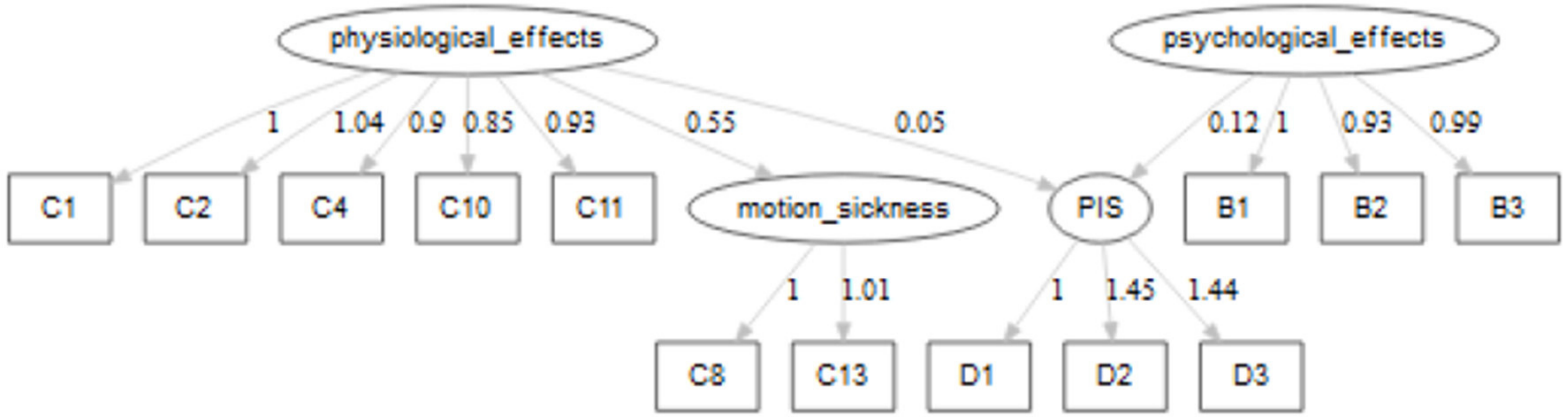
Flowchart of the SEM for the final model.

Based on the results in Table 2, the sixth model was identified as the best performing model. So before discussing the results, it is worth examining the structure of this model. It was found that the impact of “motion_sickness” latent factor would be transferred to the response through “physiological_effect,” and through that latent factor to the PIS response latent factor. However, there would be a direct relationship between “physiological_effects,” “psychological_effects,” and PIS. It was found that there were significant correlations between variables of C1 (neck pain), C2 (headache), C10 (back pain), C11 (sensation in body), and between B2 (sad) and B3 (frustrated). The following sections will discuss the relationship between latent factors and other variables. The relationship between factors and variables are depicted in Figure 3.

### Physiological_Effects

The endogenous factor of “Physiological_effects” is defined by five exogenous variables related to various physical feelings of the commuters when facing rail transport delay, and a latent factor of “motion_sickness.” The variables that made up this latent factor are somehow in line with the literature review that a range of unpleasant symptoms, such as nausea, dizziness, and headache, are common to motion sickness, and that might be due to the fact that disruption in brainstem processing might result in the evocation of a complex range of unpleasant symptoms (Cuomo-Granston and Drummond, 2010). The impacts of various exogenous variables on the factor are very similar, with the highest impact being related to C2 (headache). On the other hand, the lowest impact is related to the latent factor of motion sickness.

### Motion_Sickness

The exogenous latent factor of motion_sickness has no direct relationship with PIS, and its impact on the PIS is though the mediating factor of physiological impacts. In other words, the latent factor of motion_sickness has a contributory indirect impact on physiological latent factor. As expected, the variables of motion sickness and stomach pain have contributory impacts on this latent factor with almost identical values and signs.

### Psychological_Effects

The psychological factor includes feelings such as anger, sadness, and frustration, with all the feelings having contributory impacts on this latent factor. The results of this latent factor indicate that all the feelings have an almost identical and positive impact on the response.

### PIS

Moving to the dependent latent of this study, PIS, This latent factor's variables are all related to various measurement of PIS. The variables considered for this latent factor include whether an inaccurate PIS increases the perceived waiting time (perceived waiting time, D1), whether higher satisfaction with the rail transport system would be obtained if PIS worked properly even if the rail is delayed (satisfaction, D2), and if a correct performance of PIS increases the quality of the rail transport system (rail transport quality, D3). It should be noted that the correlations between D1, D2, and D3 were considered but they resulted in a decrease of the model fit so we let them be constrained.

The path between other latent factors and this dependent latent factor would highlight if various feelings that the commuters experience have an impact on the perceived rail quality based on the PIS. In other words, it is hypothesized that the commuters who undergo a higher degree of various psychological and physical effects due to delay assign more importance to the PIS.

The exogenous latent factors to the latent factor of PIS are psychological and physiological latent factors, with the higher impact being due to psychological_effects. Both of those factors have contributory effects on the PIS. In other words, commuters who experience a higher degree of various psychological or physiological effects of delay would assign a higher importance to the PIS.

## Discussion

Transport delay is not just about the valuation of money or time of the commuters, but also concerns the psychology of the commuters. Although delay is inevitable in many public transport systems around the world, policymakers have been doing their best to reduce the negative impact of delay on commuters by informing them about the expected waiting time or providing some forms of reimbursement. Still, many public transport systems have been suffering from extended delays, and at the same time they might provide the passengers with inaccurate expected waiting times.

This study was conducted to fulfill the following objectives: what are the various physical and psychological effects that commuters might experience when waiting for delayed rail transport? And, consequently, what is the relationship between those perceived feelings and assigned importance to the real-time information? The structure of the hypothesis could be checked by implementation of factor and SEM analyses. The SEM as a multivariate statistical analysis has been used to analyze the structural relationship of the attributes of the model. The method has been preferred to some traditional techniques due to estimation depiction of multiple and interrelated dependence of a single model. With this technique, the relationship between explanatory and latent variables and responses would be revealed. The proposed structure was first tested based on Cronbach's alpha and empirical BIC methods. The methods confirm that all the variables, assigned by factor analysis, should be kept in their factors and number of factors to be set as 4, resulting in the lowest BIC measure. After highlighting the number of factors along with the variables, various SEM models with various structures and paths were considered. We implemented various paths for the included factors to highlight the path across the latent factors and other explanatory variables.

Four factors were identified, including two factors related to physiological feelings, a factor related to psychological feelings, and a factor including three attributes related to the quality of PIS, as the response. While stomach pain and feelings of motion sickness due to delay were grouped under “motion_sickness,” five other variables, such as neck pain and headache, were categorized under physiological factor. On the other hand, three variables related to the quality of PIS were grouped under a response factor.

The identified structural results have expected signs and path directions. For instance, it was found that the higher the degree of the negative feelings that the commuters experience, the greater importance they would assign to the PIS. Also, it is important to note that the impact of the latent factor of “motion_sickness” on PIS would be mediated by the latent factor of physiological latent. In addition, it was found that the impact of physiological effects of delay on the perceived impact of PIS is higher than physiological latent factors.

### Concluding Remarks

The study evaluates the connection between the feelings of commuters due to delay and real-time information. The system would inform the commuters regarding the live departure and arrival of the transport vehicles. Consequently, the perceived uncertainty of commuters could be reduced. However, the concept relies on the fact that the system is reliable. The current study tried to connect the real-time information with feelings of the commuters due to delay and perceived quality of the transport system.

The current study has important implications for policymakers. It highlights the importance of PIS on the perceived quality of the rail transport system and how an accurate real-time information system provided for the commuters would increase the perceived quality of a rail transportation system, even if the transport is expected to be delayed. On the other hand, inaccurate real-time information would have an aggravated negative impact on the quality of the rail transport system and various perceived negative effects that the commuters might experience.

The results highlighted that, when the delay of a transport is inevitable, the policymakers, by taking small steps such as providing accurate real-time information, could provide enough information for the passengers so the negative effects of delay would be minimized. That would increase the reliability and quality of the service and, consequently, the satisfaction of the service users.

## Data Availability Statement

The raw data supporting the conclusions of this article will be made available by the authors, without undue reservation.

## Author Contributions

MR: analysis. FRF: supervision. Both authors contributed to the article and approved the submitted‘version.

## Conflict of Interest

The authors declare that the research was conducted in the absence of any commercial or financial relationships that could be construed as a potential conflict of interest.
